# Synthesis and Spectral Characterisation of Fabricated Cerium-Doped Magnesium Oxide Nanoparticles: Evaluation of the Antimicrobial Potential and Its Membranolytic Activity through Large Unilamellar Vesicles

**DOI:** 10.3390/jfb14020112

**Published:** 2023-02-17

**Authors:** Ashapurna Khatua, Kajal Kumari, Deepak Khatak, Annesha Roy, Neelima Bhatt, Bernard Paul, Aparupa Naik, Amiya Kumar Patel, Uttam Kumar Panigrahi, Santosh Kumar Sahu, Muthupandian Saravanan, Ramovatar Meena

**Affiliations:** 1 School of Environmental Sciences, Jawaharlal Nehru University, New Delhi 110067, India; 2 A.I. Virtanen Institute for Molecular Sciences, Univerisity of Eastern Finland, 70211 Kuopio, Finland; 3 Institut Jules Guyot, University of Bourgogne, 21000 Dijon, France; 4 School of Biotechnology and Bioinformatics, Sambalpur University, Odisha 757003, India; 5 Department of Physics, Maharaja Sriram Chandra Bhanja Deo University, Odisha 757003, India; 6 AMR and Nanotherapeutics Lab, Department of Pharmacology, Saveetha Dental College, Saveetha Institute of Medical and Technical Sciences (SIMATS), Chennai 600077, India

**Keywords:** cerium doped in magnesium oxide (MgOCeNPs), antimicrobial activity, membrane permeability, reactive oxygen species

## Abstract

Considerable attention has been given to Magnesium oxide nanoparticles lately due to their antimicrobial potential, low toxicity to humans, high thermal stability, biocompatibility, and low cost of production. However, their successful transformation into sustainable drugs is limited due to their low membrane permeability, which reduces their bioavailability in target cells. Herein we propose Cerium-doped magnesium oxide nanoparticles (MgOCeNPs) as a powerful solution to above mentioned limitations and are compared with MgO NPs for their membrane permeability and antimicrobial activity. Both pure and Ce-doped were characterized by various spectroscopic and microscopic techniques, in which an X-ray diffraction (XRD) examination reveals the lattice patterns for doped nanoparticles. Furthermore, Atomic Force Microscopy (AFM) revealed the three-dimensional (3D) structure and height of the nanoparticle. The crystal structure (FCC) of MgO did not change with Ce doping. However, microstructural properties like lattice parameter, crystallite size and biological activity of MgO significantly changed with Ce doping. In order to evaluate the antimicrobial potential of MgOCeNPs in comparison to MgO NPs and to understand the underlying mechanisms, the antibacterial activity was investigated against human pathogenic bacteria *E. coli* and *P. aeruginosa,* and antifungal activity against THY-1, a fungal strain. MgOCeNPs were studied by several methods, which resulted in a strong antibacterial and antifungal activity in the form of an elevated zone of inhibition, reduced growth curve, lower minimum inhibitory concentration (MIC_80_) and enhanced cytotoxicity in both bacterial and fungal strain as compared to MgO nanoparticles. The study of the growth curve showed early and prolonged stationary phase and early decline log phase. Both bacterial and fungal strains showed dose-dependent cytotoxicity with enhancement in intracellular reactive oxygen species (ROS) generation and formation of pores in the membrane when interacting with egg-phosphatidylcholine model Large Unilamellar Vesicles (LUVs). The proposed mechanism of MgOCeNPs toxicity evidently is membranolytic activity and induction of ROS production, which may cause oxidative stress-mediated cytotoxicity. These results confirmed that MgOCeNPs are a novel and very potent antimicrobial agent with a great promise of controlling and treating other microbes.

## 1. Introduction

Infectious diseases are chronic worldwide and had a critical health concern for hundreds of years, attracting significant public attention globally as a human health risk [1]. Among these, microbial infections are a major concern worldwide. The discovery and use of antibiotics, which has received great attention in recent years, has helped to treat infectious illnesses and advanced biomedicine. Widespread usage of broad-spectrum antibiotics has led to drug resistance against some human disease-causing microbes. Therefore, antibiotic resistance reduces therapeutic benefits and increases mortality. Nanomaterials’ unique antibacterial action helps us to reduce bacterial medication resistance. Recently, a range of metal oxide-based nanomaterials has been completely incorporated into antimicrobial applications and shown significant enhancement [2]. The distinct physicochemical features of nanosized materials enable the formation of tailored nanoparticles with several applications [3]. Researchers have conducted extensive studies on the controlled synthesis of metal and non-metallic nanoparticles of a specified size and form for their specific applications, such as chemical and biological sensing [4], drug delivery [5], gas sensing [6], capturing of CO_2_ [7], etc. MgO is an inorganic nanoparticle that has drawn wide-ranging attention due to its fundamental properties like as ceramics, high reactivity and specific surface area utilised in catalysis, wide band gap (7.8 eV), low dielectric constant and thermodynamic stability and is also known to have several types of intrinsic defects with Mg and O vacancies [8,9]. In addition to this non-toxicity nature of MgO nanoparticles leads to potential applications in therapeutic uses like biological labelling, band-aids, blood collecting vessels, coated capsules, etc. [10]. In bulk counterpart, MgO exhibits a cubic structure with wide band gap nature. When it is reduced to the nanoscale region, the properties of MgO drastically change. Previous studies have shown that the bactericidal activity of MgONPs increases with decreasing their size [11]. Generally, doping is one of the suitable methods to increase the surface area and narrow the bandgap of the parent material, which leads to enhancing the photocatalytic properties, antibacterial activity and absorbance properties of visible light, etc. Among the rare earth elements, Cerium (Ce) has two stable oxidation states, i.e., Ce^3+^ and Ce^4+^ and a high ionic radius (0.90 Å), which easily replaced the Mg^2+^ ion in the MgO crystal system [12]. Besides this, the nanoceria is a regenerative radical scavenger, efficiently scavenges superoxide radicals (producing H_2_O_2_), while a smaller percentage of surface Ce^3+^ is expressing peroxidase or catalase mimetic activities, such as the ability to breakdown H_2_O_2_ into water and oxygen [13]. Therefore, nanoceria provides a broad spectrum of antioxidants and has anticancer properties, as it has toxic effects on cancer cell lines, such as lung and prostate cancer cell lines [14]. It is also used to treat illnesses caused by reactive oxygen species (ROS), including Alzheimer’s disease, ageing, Parkinson’s disease, trauma, ischemic stroke, heart disease, and cancer [15]. Therefore, we have chosen Ce as the dopant material in the present work. This work may reveal plausible pathways for antibacterial activities, as well as different designs of cerium-doped Magnesium oxide nanoparticles and their antimicrobial and membrane leakage permeability potential.

## 2. Materials and Methods

### 2.1. Chemical Reagents

Magnesium nitrate (Mg(NO_3_)_2_6H_2_O), Cerous Nitrate Hexahydrate, 3-(4,5-dimethylthiazol-2-yl)-2,5-diphenyltetrazolium bromide (MTT), Triton-X-100, HEPES, N-(7-nitrobenz-2-oxa-1,3-diazol-4-yl)-1,2-dihexadecanoyl-sn-glycero-3-phosphoethanolamine, triethylammonium salt (NBD-PE), and egg-phosphatidylcholine (egg-PC)were purchased from Sigma (Sigma-Aldrich Chemicals Private Limited, Delhi, India). Potato Dexture Agar (PDA), Muller Hinton agar, Luria-Bertani Agar (LBA), Hoechst 33342, and Sodium dithionite were purchased from Hi-Media, India. All routine chemicals were purchased from Merck (India), and all chemical stock solutions were prepared in deionised distilled water (dH_2_O). All glassware and chemicals were used in analytical grade, and all experiments were properly washed twice with distilled water prior to use.

### 2.2. Maintenance of Microbes

The bacterial strains of *E. coli* (MTCC-118) and *P. aeruginosa* (MTCC-1035) were inoculated in Luria broth and were kept at 37 °C overnight to obtain an exponential growth phase to use for further study.

The THY-1 sequence was submitted to GenBank by Paul, B and Voglmayr, H (2013) under the GeneBank accession of KF806442. This strain was isolated from Nagpur, India, by Prof. Paul, where the aforementioned professor has graciously consigned it to the author. Although the oomycete strain THY-1 was named *Pythium siamicum*, it was never described as a new species. A BLAST search shows that it closely resembles *Pythium nunn* (97.74% identity, *Globisporangium nunn*). THY-1 was used for the microbial studies, and the strains were cultured in PDA slants, while fresh potato dextrose broth (PDB) was used for the inoculation process. All experiments were performed at 25 °C. Fungal species confirmation was performed using an 18s rDNA sequence. A fragment of amplified 18s rDNA was compared to the NCBI GenBank database.

### 2.3. Cerium-Doped Magnesium Oxide Nanoparticles

There are various synthesis methods available in the literature for the synthesis of powder-form MgO nanoparticles. We, however, have adopted a simple and cost-effective wet chemical method to synthesise the MgO nanoparticles. The pure and doped MgO nanoparticles of good reproducibility could be synthesised in our method. The MgO and Ce-doped MgO nanoparticles were prepared using Mg(NO_3_)_2_·6H_2_O and CeN_3_O_9_·6H_2_O as the initial precursors and an aqueous NaOH solution (0.8 M) as the precipitation agent. A stoichiometric amount of Mg(NO_3_)_2_·6H_2_O and CeN_3_O_9_·6H_2_O were mixed with distilled water at a temperature of 60 °C and 200 rpm for 4 h using a magnetic stirrer. During the stirring period, NaOH solution was added dropwise to form precipitation evaporating it under a hot plate. The resulting powder was sintered at 600 °C for 1 h to obtain MgO and MgOCeNPs nanoparticles, which were characterised before biological application.

### 2.4. Characterisation of MgOCeNPs

The nanoparticles were characterised by using various spectroscopic and microscopic techniques. In brief, the purity and crystal size of MgOCeNPs was measured by an X-ray diffractometer with a CuKα target (λ = 1.5406 Ǻ) and a Peltier-cooled solid-state detector (X-ray Diffractometer Bruker AXS Microstar TM, New Delhi, India ). The crystallite size was determined using the Debye-Scherrer relation that uses the peak position and peak full-width half maximum (FWHM) as the input parameters. Furthermore, to identify the phase and phase transition of MgOCeNPs, the Raman spectroscopy was used with the WiTec model alpha 300 having LASER of wavelength 532 nm.

The detailed structural and morphological analysis of MgOCeNPs was done using a transmission electron microscope (TEM and HRTEM analysis). Samples were drop-casted onto carbon-coated copper grids and air-dried. The grids were observed using a Transmission Electron Microscope (TEM- JEOL 2100F, New Delhi, India) equipped with a tungsten filament (200 kV). Furthermore, surface morphology, particle size and elemental mapping of MgOCeNPs were calculated by scanning electron microscopy (Scanning Electron Microscope, SEM–Zeiss EVO40, Mumbai, India) equipped with an EDX analyser. The elementary mapping analysis was performed on FESEM (FESEM with FIB and EBL Tescan Model LYRA 3 XMU, Mumbai, India). At the same time, the Atomic Force Microscopy (Atomic Force Microscopy Park XE 70, Bangalore, India) analysis was performed to reveal information about the topography of the surface and display the height of the nanoparticles. The instrument operates via an interaction between the sample and a sharp tip. The probing tip is attached to a cantilever, and samples were located using a charged coupled device monitor. This deflection is detected and mapped to scan the surface.

### 2.5. Assessment of Antimicrobial Activity

MgOCeNPs were tested for antibacterial and antifungal efficacy against Gram-negative bacteria such as *E. coli*, *P. aeruginosa*, and the oomycete THY-1. Bacterial cells were cultivated on Luria-Bertani (LB) agar at 37 °C for 24 h. The antibacterial experiment was conducted using a Muller–Hinton agar. At the same time, the oomycete was cultured on PDB at 25 °C for 24 h. The antibacterial work was carried out by a procedure reported in earlier investigations [16,17].

### 2.6. Agar Well Diffusion Method

The antibacterial potential of MgOCeNPs was evaluated using a well diffusion experiment. In order to achieve the ideal temperature, overnight-grown bacterial and fungal cells were cultivated, bacteria were grown on Luria broth, but Muller Hinton agar plates were used for agar well diffusion for easy drug release, and fungal strains were grown on Potato dextrose agar plates. 15 mL of sterile nutrient agar was poured into the sterile Petri plates; plates were inoculated with 100 µL of the fresh overnight culture (10^8^ CFU/mL) of targeted pathogenic bacteria. The above strain was supplemented with 80 µg/mL of MgOCeNPs dissolved in distilled water in the 6mm well that was previously prepared using a sterile cork borer. The plates were incubated overnight at 37 °C for bacteria and 25 °C for the oomycete. After incubation, the diameter of the inhibition zone was determined, and data were analysed according to the CLSI standard.

### 2.7. Minimum Inhibitory Concentration (MIC)

The lowest concentration of MgOCeNPs, which inhibit bacterial growth to 3-fold units, is known as Minimum inhibitory concentration (MIC) and was investigated using a common technique double dilution broth method [18]. In short, overnight suspensions of bacterial and fungal cells in 0.85% N-saline solution were made such that the absorbance was 0.1 at 600 nm. The MgOCeNPs and MgO were introduced to the first well after being serially diluted in a concentration range of 80 µg/mL. The plate was then incubated at 25 °C for fungal growth and 37 °C for bacteria in a shaker incubator for 24 h. At 600 nm, the reading was taken. The standard formula [19] was used to get the MIC_80_ value, which is the concentration at which cell death has been seen in 80% of cases. The standard deviation was computed after each experiment was carried out in triplicate.

### 2.8. Growth Curve

The growth curve was evaluated using a similar process. For this, fungal and bacterial cells were diluted in potato dextrose broth and Luria-Bertani broth, respectively [20]. Three sets of *E. coli, P. aeruginosa,* and THY-1 were treated with various doses of MgOCeNPs at 1.84 µg/mL, 9.03 µg/mL, and 11.63 µg/mL, and MgO containing 5.61 µg/mL, 23.26 µg/mL, and 14.05 µg/mL for each set, The doses decided for MIC_80_ value was the lowest concentration of the nanoparticles that did permit inhibition visible growth of the pathogenic strains, all while being maintained at the ideal temperature. At 48-h intervals of incubation, aliquots were taken, and the OD at 600 nm was noted.

### 2.9. Viability Test (MTT Assay)

In 96-well plates, cultures of bacterial and fungal strains were seeded and incubated at the appropriate temperatures. MgOCeNPs and MgO that have been optimally synthesised are both treated at concentrations of 1.3, 2.7, 5.4, and 10 µg/mL (the mean value of both particles has been considered here) and incubated for 24 h. The media was removed after the completion of incubation times, and MTT (1 mg/mL) 100 µL was added to each well. The plate was then incubated for 3 to 4 h at 37 °C for the reduction of yellow tetrazolium salt (3-4,5-dimethylthiazol-2-yl)-2,5-diphenyltertazolium bromide) to formazan [21]. Following the addition of DMSO 100 µL in each well for the solubilisation of the formazan complex, the absorbance reading was taken at 540 nm, and cell viability was calculated using the percent inhibition formula [22].

### 2.10. Reactive Oxygen Species

Additionally, DCFH-DA was used to estimate the production of intracellular ROS in bacterial and oomycete mycelium. For this, an overnight incubated bacterial culture with an OD value of 1 at 600 nm was centrifuged, and the pellet was split into three portions according to a previously standardised protocol [23,24,25]. Then, the bacterial and oomycete mycelium were treated with MgOCeNPs and MgO in a range of ratios and incubated for 4 h at 37 °C. Following the incubation period, the suspension was rinsed three times with distilled water before being added for 30 min in the dark with 10 µM DCFH-DA. PBS was used to wash the cells before readings were taken absorbance at 488 nm and 540 nm.

### 2.11. Leakage Assay

Large unilamellar vesicles (LUVs) mimic from egg-phosphatidyl choline (egg-PC) were used to quantitate the membranolytic activity of MgOCeNPs [26]. Briefly, the LUVs were prepared following the electroformation on copper electrodes using egg-phosphatidyl choline (egg-PC) that contained 0.3 molar% of fluorescent phospholipid analog N-(7-nitrobenz-2-oxa-1,3-diazol-4-yl)-1,2-dihexadecanoyl-sn-glycero-3-phosphoethanolamine and triethylammonium salt (NBD-PE). Approximately 10^3^ LUVs containing ~10 nmol phospholipids were incubated at 25 °C for 5 min and 1 mg/mL MgOCeNPs in a total volume of 200 μL in assay buffer (10 HEPES, pH 7.5 and 100 mM NaCl) in 96 well plates. Leakage induced in LUVs by MgOCeNPs was quantitated using a time-dependent fluorescence quenching of NBD-PE by the membrane-impermeable quencher sodium dithionite. The steady initial fluorescence (F_0_) was recorded using a JASCO FP-6500 spectrofluorometer (Japan). At 1 min, 10 μL of 1 M sodium dithionite made in 1 M freshly prepared Tris-base (pH 10) was added to the sample, and fluorescence intensity was monitored for 2 min. Control vesicles that were not treated with MgOCeNPs observed a fluorescence reduction by ~50%, while an increased quenching (>50%) of NBD-PE fluorescence was observed for MgOCeNP-treated vesicles due to leakage of dithionite into the LUVs. Percentage leakage activity induced by the nanoparticles was calculated from the percentage NBD-fluorescence reduction (PFR) after dithionite addition using the following formula. Percentage fluorescence reduction (PFR) = ([(F_0_ − F_f_)/F_0_]_Treated_ × 100) − ([(F_0_ − F_f_)/F_0_]_Control_ × 100)

Where F_0_ is the initial quenchable steady state of fluorescence intensity, and F_f_ is the final quenchable steady-state fluorescence of the vesicles after dithionite addition. The quenchable fluorescence for F_0_ and F_f_ was determined by subtracting the residual fluorescence left over in the sample after the addition of Triton X-100.

### 2.12. Statistical Analysis

All experiments were performed in triplicates. The mean ± SD was determined. The significance of the data was tested using two-way ANOVA followed by Tukey post hoc test using GraphPad Prism 9 software.

## 3. Results

### 3.1. Spectral Characterisation of MgOCeNPs

**Figure 1:**
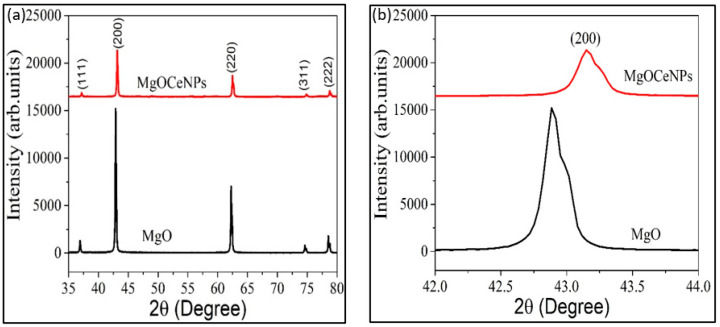
(**a**) represents the XRD pattern of MgO and MgOCeNPs, and (**b**) illustrates the view of 200 peaks of MgO and Ce-doped MgO samples.

In order to confirm the synthesis of magnesium oxide nanoparticles, all the parameters of characteristics points support the synthesis of MgOCeNPs. XRD pattern of MgO and MgOCeNPs shows that all the peaks present in the spectra are related to MgO, indicating face-centred cubic (FCC) with a space group of Fm3m with no secondary peak was observed in the spectra designating that Ce is successfully substituted in MgO (Figure 1A). Moreover, the results illustrate the expanded view of 200 peaks of MgO and Ce-doped MgO samples (Figure 1B). It is observed that the major peak at 200 shifted to a high diffraction angle, which further confirms that Ce was successfully substituted in the MgO. Previous studies also mentioned that shifting the diffraction angle towards a higher angle indicated that Ce doping had induced lattice contraction [27,28].

The average crystallite size (D) of MgO and MgOCeNPs was calculated using the Debye-Scherrer relation

(1) $$D=\frac{0.9\lambda }{\beta cos\theta }$$

where, *λ* = 1.54 Å = wavelength of X-ray, *β* = Full width half maxima, and *θ* = Braggs angle. The *D* value for MgO nanoparticles is 44.70 nm, and the same decrease to 36.62 nm for Ce-doped MgO nanoparticles. The decrease in particle size is also reported by another study [29] for Ce-doped MgO nanoparticles, and the authors revealed that the size of Ce-doped MgO nanoparticles decreases from 35 to 23 nm with increasing Ce doping concentration from 0% to 5%. The strain (*ε*) of the samples is calculated by the following relation (Equation (2)):

(2) $$\varepsilon =\frac{\beta cos\theta }{4}$$

The calculated strain for MgO and MgOCeNPs is 7.76 × 10^−4^ and 9.95 × 10^−4^, respectively. The decrease in crystallite size in Ce-doped samples is explained as follows. The ionic radius of Ce^4+^(0.90 Å) is larger than Mg^2+^ (0.72 Å), and hence, Ce doping has induced additional strain in the lattice. The Ce^4+^ ions were accommodated on the crystallite surface to minimise the strain. This led to an increase in volume ratio, which is essential to reduce the crystallite. The lattice parameter (*a*) for cubic FCC lattice can be calculated as follows:

(3) $$\frac{4sin^{2}\theta }{\lambda^{2}}=\frac{1}{d^{2}hkl}=\frac{h^{2}+k^{2}+l^{2}}{a^{2}}$$

**Figure 2:**
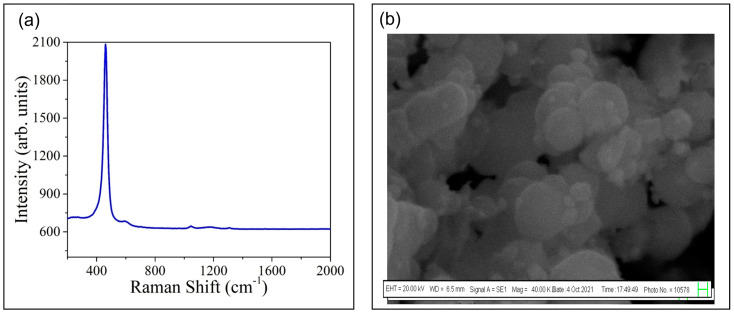
(**a**) Raman spectra of MgOCeNPs; and (**b**) Scanning Electron Microscope of MgOCeNPs. The obtained image shows that particles are spherical in nature with little agglomeration.

The value of the lattice parameter for MgO and MgOCeNPs is 4.21Å and 4.18 Å, respectively, which also confirmed that Ce doping in MgO has induced contraction of lattice constants. Raman spectra of Ce-doped MgO (MgOCeNPs) nanoparticles ranged from 200 to 1500 cm^−1^. The MgO characteristic peak present at ~449 (major) and 1305.603 cm^−1^ is deemed promising according to the reported literature [30,31,32,33]. These types of bands are not observed in bulk MgO, which indicates the prepared sample is crystalline in nature; this result is consistent with the XRD analysis. Results show the band at ~1041 cm^−1^ attributed to TO + LO surface phonon mode present in the samples in this study, whereby Raman’s study again confirmed that Ce doping in MgO did not induce any impurity phase related to Ce or CeO (Figure 2a). Meanwhile, the surface morphology of MgOCeNPs, as shown in SEM images, illustrates that the particles are spherical with low agglomeration (Figure 2b).

**Figure 3:**
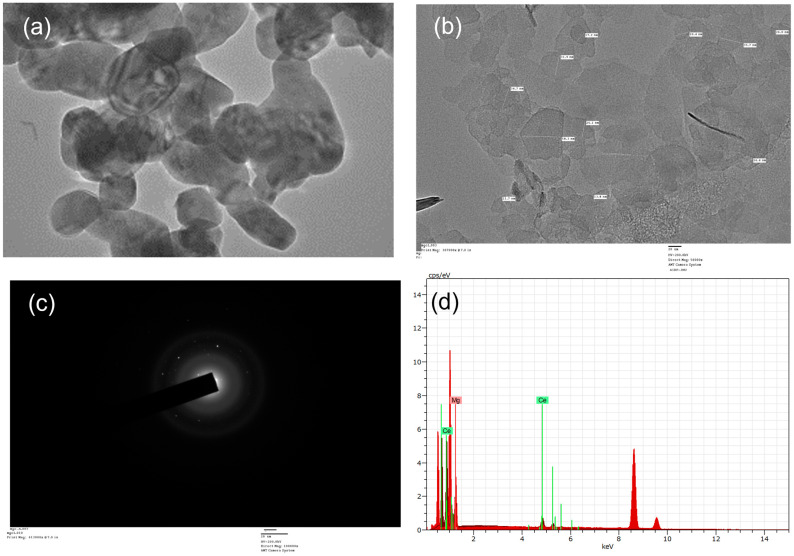
(**a**) High Resolution Transmission electron microscope (HRTEM) demonstrated that particles are spherical in nature with existence of agglomeration; (**b**) Transmission electron microscope (TEM); (**c**) Selected Area Electron Diffraction (SAED); and (**d**) EDX analyser number of counts.

**Figure 4:**
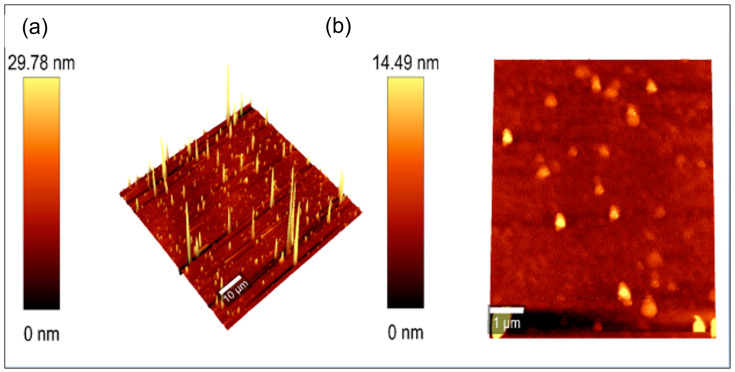
Atomic force microscopy image at (**a**) 10 µm; and (**b**) 1 µm.

Furthermore, the particle size and structure of the MgOCeNPs were determined by High resolution transmission electron microscopy (HRTEM) using 200 kV accelerating voltage; the images, Figure 3a show that the particles were hexagonal in shape. Figure 3b shows MgOCeNPs had an average size of 34.66 nm with a higher amount of polydispersity, which is quite different from the monodispersed MgO with an average size of 36.05 nm in previous studies [34,35]. At the same time, in the Figure 3c, the SAED images showed a clear lattice pattern with an interspacing for magnesium and cerium particles [36]. Due to wide variation in particle size and morphology, the SAED images showed the existence of two crystal lattices, which are in the core and the surrounding region [37,38]. Whereas the line mapping and EDX analysis,Figure 3dsuggested that the elemental composition of the nano doped confirmed the presence of Mg and Ce. The surface roughness of the MgOCeNPs was determined by atomic force microscopy (AFM), where Figure 4a,b represent two-dimension AFM images and the corresponding size distribution of MgOCeNPs. Atomic force microscope results show that the average surface diameter of MgOCeNPs is 0 to 29 nm at 10 µm and 0 to 14.49 nm at 1 µm. All physicochemical characteristics support the formation of cerium-doped in magnesium oxide nanoparticles.

### 3.2. Antimicrobial Activity of Synthesised MgOCeNPs

A plethora of antibacterial and antifungal analyses as a circle of the zone, growth curve, minimum inhibitory concentration (MIC) and cytotoxicity assays (MTT). The nanodoped MgOCeNPs were assessed for antibacterial activity against human pathogenic bacteria, *E. coli* and *P. aeruginosa*, as well as plant-damaging fungus, THY-1, in which MgOCeNPs were employed to assess the antimicrobial potential. As a result, determining the comparative potential of MgOCeNPs and MgO as antibacterial agents is critical.

**Figure 5:**
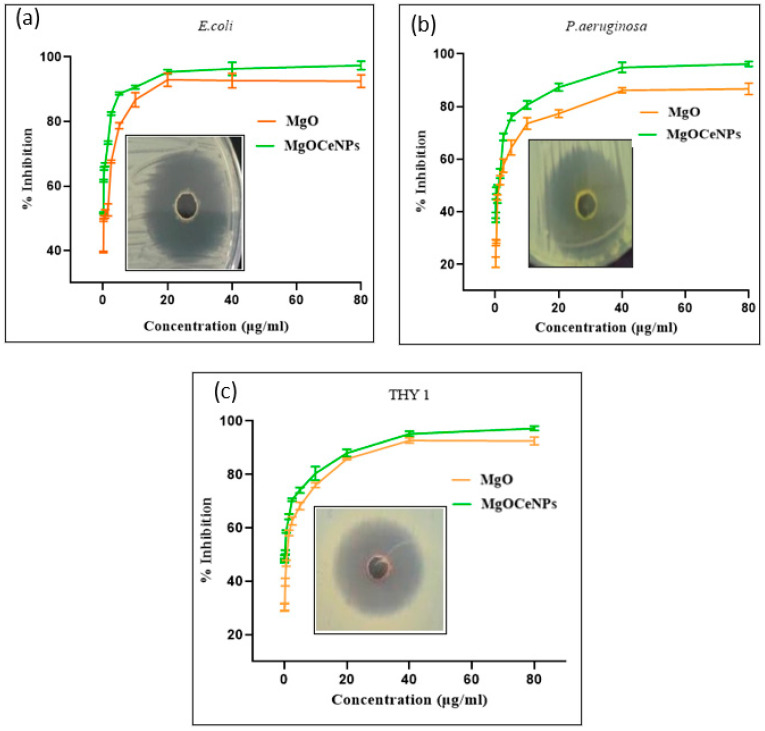
Zone of inhibition and minimum inhibitory concentration (MIC): (**a**) *E. coli*; *(***b**) *P. aeruginosa*; and (**c**) THY-1 (*Pythium siamicum*).

To assess the antibacterial and antifungal properties, serial dilution procedures were used. The well diffusion experiments revealed a distinctive zone of inhibition. MgOCeNPs have been shown to be hazardous to these bacterial and fungal strains. The results show zone of inhibition diameters of MgOCeNPs was 37 mm, 38 mm, and 33 mm, respectively, for *E. coli*, *Pseudomonas aeruginosa*, and THY-1, which is significantly higher than the zone of inhibition of MgO (Figure 5), which might be attributed to the high-affinity association between the gram-negative bacterial surface and the positively charged nanoparticles [39]. At the same time, the minimum inhibitory concentration (MIC), growth curve, MTT, and ROS levels were calculated to understand the underlining mechanism of doped nanoparticles. Meanwhile, typical microdilution tests were used to assess the antibacterial properties of MgOCeNPs and MgO. As demonstrated in Figure 5, the MIC test indicates dose-dependent toxicity, with MgOCeNPs being the most toxic to microorganisms (a, b, c). Minimum Bactericidal Inhibition and Minimum Fungicidal Inhibition of *E. coli*, *P. aeruginosa*, and THY-1 have shown MIC_50_ and MIC_80_ values in a distinct range against both nanoparticles, as indicated in Table 1.

**Figure 6:**
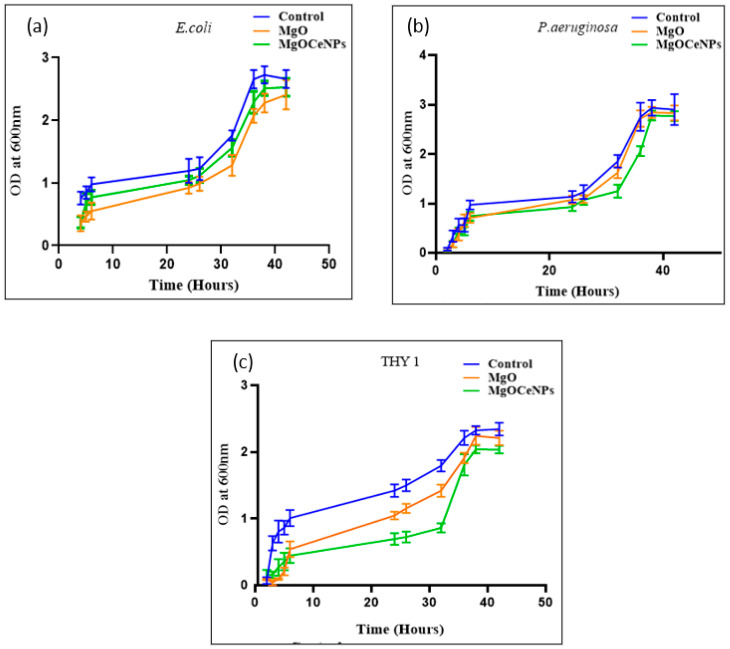
Growth curve of both bacterial and fungal strains in the decline stage: (**a**) *E. coli*, (**b**) *P. aeruginosa*; and (**c**) THY-1 (*Pythium siamicum*).

Growth curve analysis with the MgOCeNPs revealed that bacterial growth was reduced relative to control cells cultivated in LB broth and PDB for fungus growth. The growth curves of the control sets displayed obvious lag, log, and stationary phases as a function of time; however, treatment with MgO and MgOCeNPs resulted in a progressive shortening of the log phase (Figure 6). Arrested cell development was reported by nanoparticles in the exponential phase of growth, which is essentially extended therapy in the lag phase. This may be because bacterial cells take longer to adapt to their surroundings. As a result, these elements may lead to a decrease in bacterial and oomycete linear growth rates after treatment.

**Figure 7:**
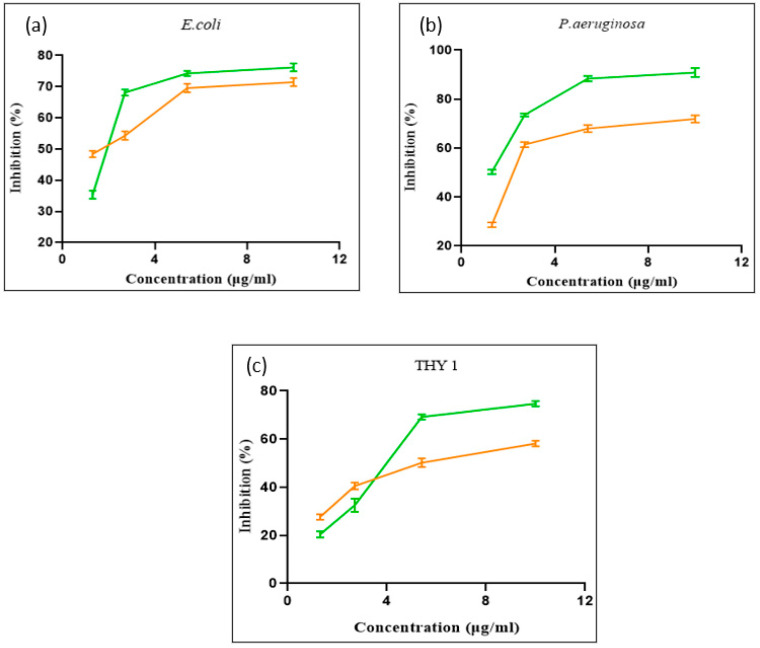
Cell viability of (**a**) *E. coli*, (**b**) *P. aeruginosa*, and (**c**) THY-1 against different concentrations of MgOCeNPs and MgO.

In addition to the above experiments, an MTT assay was performed to determine the dose-dependent toxicity of MgOCeNPs and MgO. The synthesised MgOCeNPs show higher cytotoxicity as compared to MgO. The results show the different concentrations of MgOCeNPs and MgO at which 80% of cell death values were observed (Figure 7), which emphasises the degree of declining point and the cram work on antibacterial (*E. coli and P. aeruginosa*) and antifungal properties (THY-1). A similar kind of trend was also observed that MgOCeNPs show the highest antibacterial potential against gram-negative bacteria *P. aeruginosa*.

### 3.3. Production of Reactive Oxygen Species (ROS)

**Figure 8:**
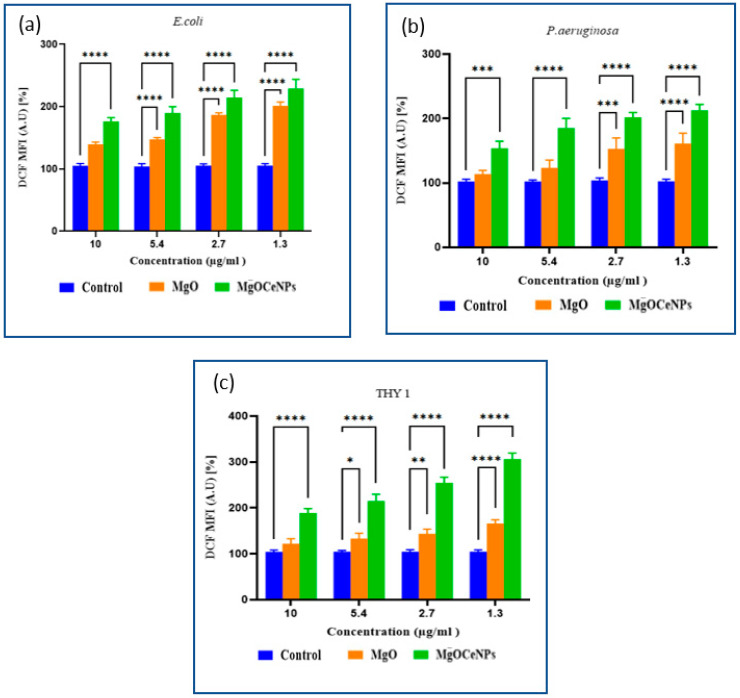
Reactive oxygen species of antimicrobial cells: (**a**) *E. coli*; *(***b**) *P. aeruginosa*; and (**c**) THY-1 *(Pythium siamicum*). * *p* < 0.05; ** *p* < 0.01; *** *p* < 0.001; **** *p* < 0.0001.

Reactive oxygen species estimation suggested a significant increase in DCFDA intensity as compared to control sets. Both MgOCeNPs and MgO NPs show a dose-dependent elevation in ROS levels in bacterial and fungal cells, but the intensity of ROS induction is significantly higher in MgOCeNPs treated cells as compared to MgO NPs (Figure 8). One more interesting result is that the intensity of ROS was comparatively higher in fungal cells as compared to bacterial cells. ROS can cause double-stranded breaks in DNA by oxidation. These antimicrobials often target redox defences. ROS accumulation leads to the damaging of biomolecules such as lipids, proteins and nucleic acid. The literature signifies that nanoparticles are directly attached to the bacterial cell surface, or their reduction leads to the release of ions, which disrupts the cell membrane and generates ROS by lipid peroxidation [40,41].

### 3.4. Phylogenetic Tree of Globisporangium Nunn THY-1 (Pythium siamicum)

**Figure 9:**
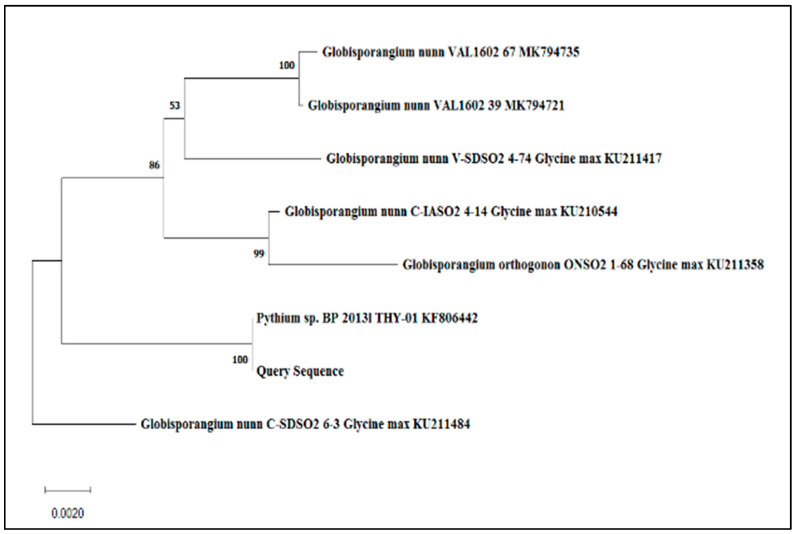
Represents the relation of the phylogenetic tree.

The ITS region of the fungal strain has been compared with the other fungal strain, which was retrieved from the NCBI GenBank using BLASTN. The multiple sequence alignment was performed using CLUSTALW, which revealed 100% identity with the sequences from strains of *Pythium* sp. BP 2013l THY-01 KF806442 and was, therefore, designated as *Pythium* sp. The phylogenetic tree was constructed using the neighbour-joining method via the software MEGA 5.2. A bootstrap test with 500 replicates was done to test the reliability of the tree, where bootstrap values of 59% or greater are indicated on the tree. The scale bar indicates the number of nucleotide substitutions per site. The phylogenetic relationship is depicted in Figure 9.

Sequences 1–5 = 18S gene partial sequence, sequences 6–204 = ITS 1complete sequence sequences 205–354 = 5.8 gene complete sequence sequences 355–888 = ITS 2, complete sequence 889–928 = 28S gene partial sequence

This sequence was submitted to GenBank by Paul, B. and Voglmayr, H. (2013) under the GenBank accession: KF806442. This strain was isolated from Nagpur, India, by Prof. Paul and was graciously handed over to us. Although the oomycete strain THY-1 was named *Pythium siamicum* by Prof Paul has not yet been described as a new species. A BLAST search shows that it closely resembles *Pythium nunn* (97.74% identity, *Globisporangium nunn*). Hence in this work, we are taking this strain as *Pythium nunn* strain THY-1. Since the globose sporangium-producing species are now grouped in the genus *Globisporangium*. The name of the oomycete thus becomes *Globisporangium nunn.*

### 3.5. MgOCeNPs Induced Leakage in LUV Model Membrane

**Figure 10:**
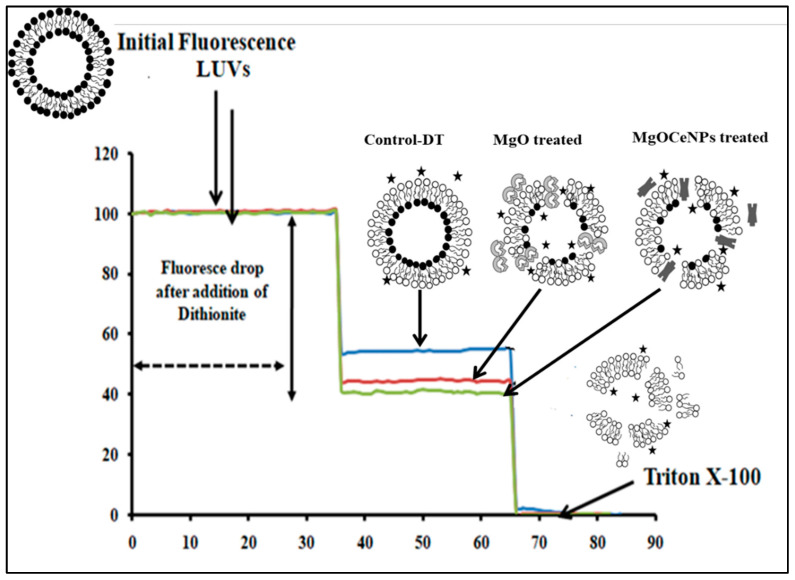
NBD-fluorescence quenching assay showing increased leakage of LUV membranes induced by MgOCeNPs. The blue, red and green curves indicate the residual fluorescence left in the LUVs of control (untreated), MgO-treated and MgOCeNP-treated samples, respectively, after the NBD-fluorescence in the outer membrane is quenched by the membrane-impermeable quencher sodium dithionite. The star represents sodium dithionite.

MgOCeNP-induced leakage in the LUV membrane was quantitated by measuring the percentage reduction of NBD-fluorescence by membrane-impermeable fluorescence quencher sodium dithionite. In control vesicles, fluorescence reduction after dithionite addition was ~50% of initial fluorescence. However, for MgOCeNP-treated LUVs, the fluorescence reduction was 60% due to the nanoparticle-induced leakage of dithionite into the lumen of LUVs that quenches NBD-fluorescence localised to the inner leaflet (Figure 10). This result indicates that the MgOCeNPs interacted with the LUV membrane, which resulted in pore formation. Treatment with triton X-100 led to complete lysis of LUVs that enabled the membrane-impermeable dithionite to access the NBD-Pes localised to the inner membrane leaflet, which resulted in almost complete (~100%) fluorescence reduction. MgOCeNP-treated LUVs showed 20% enhanced lysis of LUVs indicated by increased quenching of fluorescence. These results indicate that MgOCeNPs exhibit membranolytic activity on LUVs; the same mechanism may be worked in bacterial cells, and reactive oxygen species-mediated membranolytic activity may be the main cause of cytotoxicity.

## 4. Discussion

There has been a significant amount of investigation on inorganic antibacterial nanoparticles, more especially the oxides of metals such as titanium, zinc, magnesium, and calcium. What has been found is that these nanomaterials can resist extreme conditions and can be used by humans without risk [42,43,44]. Researchers have found that magnesium oxide nanoparticles with diameters ranging from ~10 to 60 nm have the potential to be used in the fields, but their applications are limited due to their low membrane permeability, which reduces their bioavailability in target cells. Therefore, doping is one of the suitable methods to increase surface area and narrow the bandgap of the parent material, which leads to enhancing the photocatalytic properties, antibacterial activity and membrane permeability [43]. In order to obtain this, cerium-doped magnesium oxide nanoparticles were synthesised and further characterised. XRD results show that the major peak at 200 shifted to a high diffraction angle, which confirms that Ce was successfully substituted in the MgO. Meanwhile, Raman’s study further confirmed that Ce doping in MgO did not induce any impurity phase related to Ce or CeO. The size calculated from XRD of MgO nanoparticles was 44.70 nm, which further decreased to 36.62 nm in Ce-doped MgO nanoparticles, which is in line with previous studies. At the same time, the strain calculated from XRD shows a declining trend, which may be due to the ionic radius of Ce^4+^ (0.90 Å) being larger than Mg^2+^ (0.72 Å), and hence, Ce doping has induced additional strain in the lattice. The Ce^4+^ ions were accommodated on the crystallite surface to minimise the strain. MgOCeNPs had an average size of 36.62 nm with a higher amount of polydispersity, which is quite different from the monodispersed MgO with an average size of 44.70 nm in previous studies [44,45]. All physicochemical characteristics support the formation of cerium-doped in magnesium oxide nanoparticles.

As a consequence of their antibacterial and antifungal properties, they have emerged as potential ingredients in the development of antibacterial and antifungal drugs, as well as in pharmaceutical products. In previous studies, only limited experiments were carried out in order to evaluate the antibacterial and antifungal capabilities of MgONPs on a wide variety of bacterial and fungal strains, which would not provide sufficient evidence of their antimicrobial potential. The particular action mechanism that is responsible for the antibacterial characteristics of MgOCeNPs is still a topic of inquiry at this point in time. However, in order to understand how MgOCeNPs influence various kinds of bacterial strains, only a few distinct approaches were applied [46]. Therefore more detailed experimental studies are performed to understand the antimicrobial mechanism.

The results of our present study used reactive oxygen species (ROS) as a key point to state a relationship between the nanoparticles and the bacteria. The interaction of nanoparticles with the bacterial cell wall brings about its destruction. Therefore, it is plausible to postulate that the interaction of nanoparticles with cells resulted in the creation of cellular ROS because it triggers a chain reaction of activities that included peroxidation and disruption of the cellular structure. The production of reactive oxygen species (ROS) may result in the destruction of proteins and the disruption of the enzymatic system of bacterial cells, both of which have the potential to disturb the normal physiology of bacteria [47]. The antimicrobial potential of MgOCeNPs in comparison to MgO NPs is assessed against human pathogenic bacteria, *E. coli* and *P. aeruginosa*, as well as plant damaging oomycetes, THY-1, by various experiments such as the circle of the zone, growth curve, minimum inhibitory concentration (MIC), cytotoxicity assays (MTT), and reactive oxygen species (ROS). The results of this show that MgOCeNPs significantly enhance the production of ROS, which may cause antimicrobial effects as observed by an elevated zone of inhibition, lower minimum inhibitory concentration, higher rate of cytotoxicity, arrested the cell growth in the lag phase, which may result in a progressive shortening of log phase as compared to control and MgO NPs treated cells. This shows that MgOCeNPs have strong antimicrobial potential in comparison to MgO. The scientific literature also demonstrates that MgOCeNPs have an antimicrobial potential because it inhibits the normal development of bacteria through a microbiostatic impact that is caused by the formation of reactive oxygen species (ROS) such as superoxide radicals, hydroxide radicals, and singlet oxygen [48]. The principal ways of interaction between nano-doped cells and bacterial cells are electrostatic attraction, van der Waals force, hydrophobic contacts, and receptor-ligand interactions [49,50]. After being taken into the cell, they will attach to various cellular components, which will result in a chain of reactions, including increased cell permeability, electrolyte imbalance, enzyme activity inhibition, protein inactivation, and changed gene expression. Previous studies have shown that iron oxide nanoparticles have the capacity to penetrate cells, where they could destabilise the cell’s transmembrane electrons and impair the normal functioning of the cell’s metabolism [51]. In this study, we have shown that MgOCeNPs are a highly promising option for use in the battle against the hazards presented by multidrug resistance. Their outstanding capabilities make them an excellent choice for this application (MDR). Because of the connection between the electrostatic forces on the surface of the bacteria and the MgOCeNPs, it was discovered that cell death occurred as a consequence of this contact between MgOCeNPs and bacteria [52].

Another hypothesis speculated that magnesium oxide nanoparticles are able to demonstrate their antimicrobial activity against *C. jejuni*, in which membrane leakage is one of the primary mechanisms [53]. Magnesium oxide nanoparticles were able to exert their antimicrobial action against *C. jejuni* by inducing membrane leakage. The antibacterial activity of MgO nanoparticles was revealed to be equivalent when tested against the plant pathogen *Ralstonia solanacearum* [54]. According to the results of this research, MgO NPs were to be accounted for the leakage that occurred in the very small unilamellar vesicles. Previous studies have also shown that a wide variety of compounds, including proteins, phytochemicals, and metal oxide nanoparticles, are capable of causing membrane lysis [55]. Membranolytic chemicals are very cytotoxic because they induce the lysis of the plasma membrane, which in turn allows the contents of the cell to leak out. This chain reaction is what makes these compounds so dangerous to cells. However, the permeability of cells to nanoparticles is largely regulated not only by the lipid content of the membrane but also by the surface charge and the receptors on the cell surface [56,57]. In the present study, we have found that MgOCeNPs exhibit membranolytic activity on LUVs. The MgOCeNPs significantly increased the ROS level, which is responsible for lipid peroxidation. Therefore, from the results of this study, it can be speculated that MgOCeNPs may induce ROS, which may cause lipid peroxidation that leads to damaging bacterial membranes and therefore increase the permeability of nanoparticles resulting in cell death due to excessive ROS, consequently.

## 5. Conclusions

It has been found that the formation of MgOCeNPs has strong antimicrobial potential against human pathogenic bacteria and plant pathogenic fungi. These findings have shown the mechanism of antimicrobial activity, in which the interaction of nanoparticles with bacterial cell may induce ROS production, which may cause lipid production and plays a significant role in cell death. The results study also showed that doping of Cerium on MgO NPs might elevate its antimicrobial potential in comparison to MgO NPs. Nanotechnology should be the primary focus of future research because this study demonstrated considerable toxicity of nanoparticles against pathogenic gram-negative bacterial cells as well as fungal cells, even at extremely low doses. The results of this study open up new doors for the use of multifunctional nanodoped potential bio labels in the fields of diagnostics and antimicrobials. Because of this, the biocompatibility of these nanoparticles may be explained in more depth, and judgments about the dangers that they represent to human health can be codified.

## Figures and Tables

**Table 1:** Minimum Inhibitory Concentration of MgO and MgOCeNPs against antimicrobial strains.

Strain Name	MgO	MgOCeNPs
	*MIC*_80_ (µg/mL)	*MIC*_80_ (µg/mL)
*E. coli*	5.61	1.84
*P. aeruginosa*	23.26	9.03
THY-1	14.05	11.63

## Data Availability

Data included within this article.
